# Investigation of the Effects of Saffron on Neuroprotection and Circadian Rhythm in an *In Vitro* Parkinson’s Model

**DOI:** 10.3390/ph19050773

**Published:** 2026-05-15

**Authors:** Ayse Aksoy, Duygu Deniz Usta, Atiye Seda Yar

**Affiliations:** Department of Medical Biology and Genetics, Faculty of Medicine, Gazi University, 06500 Ankara, Türkiye; ayse.aksoy5519@gmail.com (A.A.); denizsalimi@gazi.edu.tr (D.D.U.)

**Keywords:** Parkinson’s disease, L-Dopa, safranal, SH-SY5Y, 6-OHDA

## Abstract

**Background/Objectives:** Parkinson’s disease (PD) is a progressive neurodegenerative disorder characterized by dopaminergic neuronal loss, oxidative stress, and mitochondrial dysfunction. Although levodopa (L-Dopa) remains the main symptomatic treatment, prolonged administration can lead to adverse effects. Safranal, a bioactive constituent of *Crocus sativus*, has antioxidant and anti-apoptotic properties. This study evaluated the neuroprotective potential of L-Dopa and safranal, individually and in combination, in an *in vitro* cell-culture PD model. **Methods:** SH-SY5Y human neuroblastoma cells were treated with 6-hydroxydopamine (6-OHDA, 50 µM) to induce cytotoxicity. Cells were pretreated with L-Dopa (5–500 µM) and safranal (1–500 µM and 1–5 mM) for 4 or 24 h. Cell viability was assessed using 3-(4, 5-dimethylthiazol-2-yl)2,5-diphenyl-tetrazolium bromide (MTT) and lactate dehydrogenase (LDH) assays. Mitochondrial membrane potential (MMP), caspase-3/7 activity, and autophagy markers were also evaluated. Synergy was analyzed using Combination Index (CI) analysis. Furthermore, mRNA levels of circadian rhythm associated genes were also evaluated. **Results:** 6-OHDA significantly impaired cell viability and mitochondrial function. Pretreatment with low doses of L-Dopa and safranal partially improved cell viability and reduced apoptosis and showed a tendency to decrease autophagy-associated marker levels. Higher L-Dopa concentrations caused mild cytotoxicity, while high-dose safranal exhibited pronounced concentration-dependent toxicity. CI analysis confirmed synergistic interaction between both drugs in mitigating 6-OHDA-induced toxicity. Combined treatment markedly improved cell survival preserved mitochondrial function, and reduced caspase-3/7 activity compared with monotherapy. A significant increase in the mRNA levels of *Per1, Clock*, *Bmal1* and *Cry1* genes was observed in groups treated with L-Dopa and safranal together. **Conclusions:** L-Dopa and safranal exerted concentration-dependent neuroprotective effects in SH-SY5Y cells. Their combination enhanced cytoprotection, which was associated with modulation of mitochondrial function, oxidative stress, apoptosis, and autophagy-related responses.

## 1. Introduction

Parkinson’s disease (PD) is a chronic and progressive neurodegenerative disorder characterized primarily by the degeneration of dopaminergic neurons in the substantia nigra, resulting in motor and non-motor impairments [1,2]. The pathogenesis of PD involves intricate interactions between genetic predisposition and environmental triggers, with oxidative stress and mitochondrial dysfunction playing pivotal roles in neuronal death [3]. Current therapeutic strategies primarily focus on symptom management, with dopamine replacement therapy being the most effective, particularly levodopa (L-Dopa) [4]. However, long-term L-Dopa administration is often associated with adverse effects such as motor fluctuations and dyskinesia [5]. This underscores the importance of adjunctive therapies focusing on neuroprotection and disease modification [6].

Disruption of the circadian rhythm has emerged as a contributing factor in the pathophysiology of PD, affecting motor control, sleep quality, and overall quality of life [7]. Altered circadian gene expression and dysregulated melatonin secretion in patients with PD suggest a bidirectional relationship between circadian dysfunction and neurodegeneration, potentially providing new therapeutic targets [2].

Recent advances have highlighted the therapeutic potential of phytochemicals for the treatment of neurodegenerative disorders [8]. Saffron (*Crocus sativus*) has demonstrated promising antioxidant and anti-inflammatory effects, suggesting its potential to modulate neurodegenerative processes [9]. Safranal, a major bioactive component of saffron, has shown neuroprotective properties in preclinical studies by reducing oxidative damage and regulating mitochondrial function as well as inflammatory responses. These properties make safranal a promising candidate for adjuvant therapy in PD; however, further investigation is required [10].

There is growing interest in combining circadian rhythm regulation with neuroprotective strategies to improve PD management [2]. Therapies that normalize melatonin levels, optimize light exposure, or regulate sleep–wake cycles can be used alongside pharmacological agents to alleviate motor and non-motor symptoms [11]. Owing to its multifaceted biological effects, safranal could be incorporated into such synergistic approaches alongside established agents such as L-Dopa [12].

The neurotoxin 6-hydroxydopamine (6-OHDA) is widely used to model PD in experimental systems because of its selective toxicity toward dopaminergic neurons, which effectively recapitulates the pathological features of the disease [13]. 6-OHDA induces oxidative stress and mitochondrial dysfunction, ultimately leading to apoptosis [14]. *In vitro* models, particularly those using SH-SY5Y neuroblastoma cells, provide a robust platform for investigating the molecular mechanisms underlying PD and evaluating the efficacy of potential therapeutics [15]. These models facilitate the detailed investigation of oxidative injury, mitochondrial instability, and dysregulated autophagy, all of which are key contributors to dopaminergic neurodegeneration [16,17,18].

In this study, we investigated the neuroprotective effects of L-Dopa and safranal, both individually and in combination, using a 6-OHDA-induced PD model in SH-SY5Y cells. The experimental design involved treating cells with various concentrations of drugs at specific time intervals to evaluate effects on cellular viability, cytotoxicity, apoptotic and autophagic responses, and mitochondrial membrane potential (MMP), in addition to potential effects on circadian clock-related gene expression.

## 2. Results

### 2.1. Effects of 6-OHDA on Cell Viability in SH-SY5Y Cells

**Figure 1:**
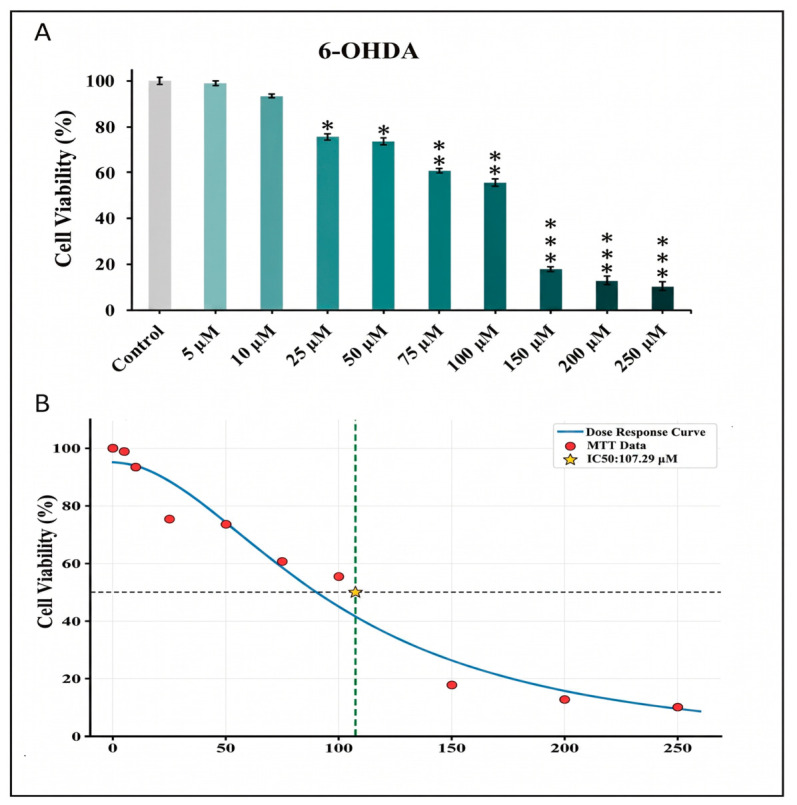
Effects of 6-OHDA on SH-SY5Y cell viability. (**A**) Concentration-dependent reduction in cell viability following 24 h exposure to 6-OHDA. The data is presented as mean ± standard deviation (SD) (*n* = 6). Statistical significance was evaluated using one-way analysis of variance (ANOVA) followed by Dunnett’s post hoc test for comparisons against the control group. Differences compared with the control group are indicated as * *p* < 0.05, ** *p* < 0.01, and *** *p* < 0.001. (**B**) Concentration–response curve of 6-OHDA in SH-SY5Y cells. The half-maximal inhibitory concentration (IC_50_) was calculated using CompuSyn software. The data is presented as mean ± SD. 6-OHDA, 6-hydroxydopamine.

Exposure of SH-SY5Y cells to 6-OHDA (5–250 µM, 24 h) resulted in a concentration-dependent decrease in cell viability, as determined by the MTT assay (Figure 1A). A significant reduction in viability was observed at concentrations ≥ 25 µM compared to the control group (*p* < 0.05), while higher concentrations (75–100 µM) induced approximately 40% cytotoxicity. Based on these results, 50 µM 6-OHDA was selected for subsequent experiments. Dose–response analysis using the Chou–Talalay method yielded the half-maximal inhibitory concentration (IC_50_) value of 107.29 µM (Figure 1B).

### 2.2. Effects of L-Dopa and Safranal Alone Without 6-OHDA on Cell Viability in SH-SY5Y Cells

**Figure 2:**
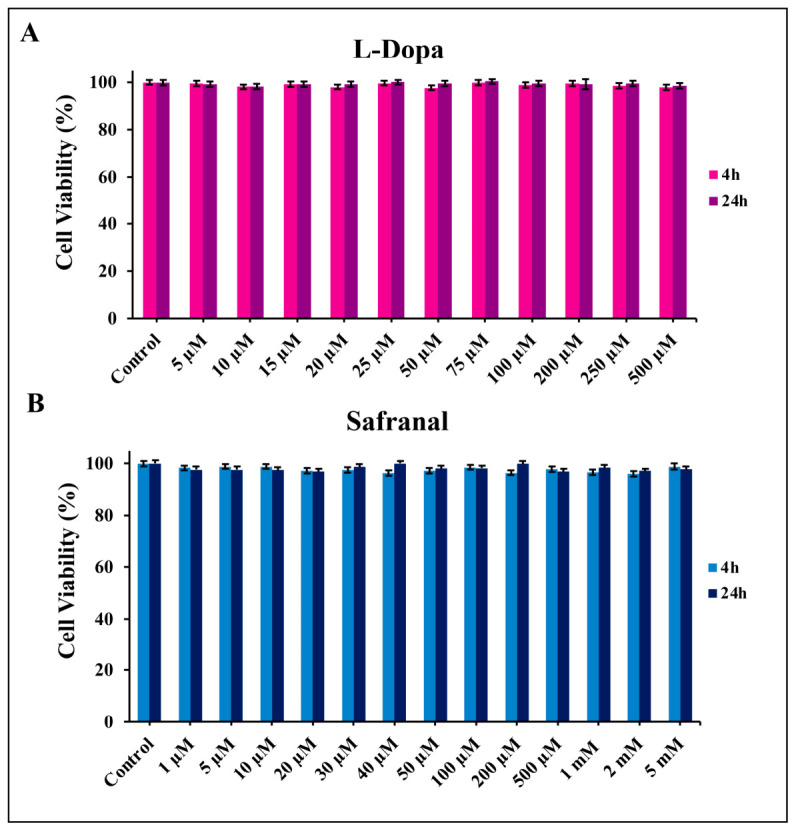
Effects of (**A**) L-Dopa and (**B**) safranal on SH-SY5Y cell viability at 4 h and 24 h. Data are presented as mean ± SD (*n* = 6). Statistical analysis was performed using two-way ANOVA followed by Tukey’s post hoc test for multiple comparisons. L-Dopa, levodopa.

The effects of L-Dopa and safranal, when administered alone, on the viability of SH-SY5Y cells were evaluated using the MTT assay. The cells were treated with increasing concentrations of L-Dopa (5–500 μM) and safranal (1–500 μM and 1–5 mM) for 4 and 24 h, respectively. Higher concentrations of L-Dopa and safranal did not have significantly stronger effects on cell viability than those seen in the control group (*p* > 0.05; Figure 2).

### 2.3. Effects of L-Dopa and Safranal, Alone and in Combination, on 6-OHDA-Induced Cell Viability in SH-SY5Y Cells

**Figure 3:**
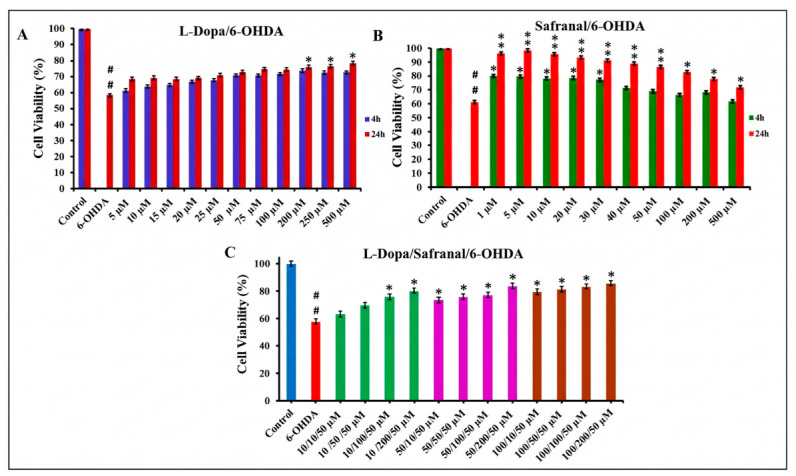
Effects of pre-treatment with (**A**) L-Dopa, (**B**) safranal, and (**C**) their combination for 4 h and 24 h, followed by 24 h exposure to 6-OHDA (50 µM), on SH-SY5Y cell viability. The data is presented as mean ± SD (*n* = 6). Statistical analysis was performed using two-way ANOVA followed by Tukey’s post hoc test for multiple comparisons. Differences compared with the control group are indicated as ## *p* < 0.01, whereas differences compared with the 6-OHDA-treated group are indicated as * *p* < 0.05, ** *p* < 0.01.

The effects of L-Dopa and safranal on cell viability following 6-OHDA-induced neurotoxicity were evaluated in SH-SY5Y cells (Figure 3). 6-OHDA significantly reduced cell viability compared to the control group (*p* < 0.01).

L-Dopa increased cell viability in a concentration- and time-dependent manner. Significant increases were observed at 200, 250, and 500 µM following 24 h pretreatment (*p* < 0.05; Figure 3A). Safranal increased cell viability in a concentration-dependent manner. The highest cell viability was observed at 1–50 µM following 24 h pretreatment (*p* < 0.01), whereas lower values were observed at 1–40 µM for 4 h and 100–500 µM for 24 h (*p* < 0.05; Figure 3B).

The effects of combined L-Dopa and safranal treatment resulted in higher cell viability compared to the 6-OHDA group (*p* < 0.05). The highest cell viability was observed in cells treated with 100 µM L-Dopa and 10 µM safranal under 4 h pretreatment conditions (Figure 3C).

**Figure 4:**
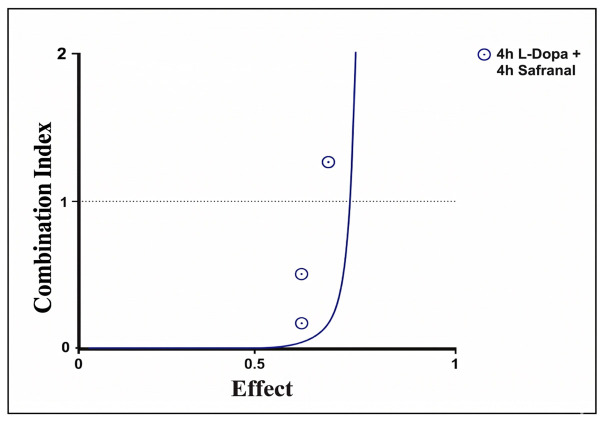
Combination index (CI) analysis of the interaction between levodopa and safranal. CI values were calculated using the Chou–Talalay method implemented in CompuSyn software. The plotted curves represent CI values across a range of effect levels (Fa, fraction affected; 0–1, corresponding to 0–100% inhibition). CI < 1 indicates synergism, CI = 1 indicates an additive effect, and CI > 1 indicates antagonism.

Combination Index (CI)–fraction affected (Fa) analysis was performed to evaluate the effect of the L-Dopa and safranal combination on SH-SY5Y cells. Following short-term (4 h) pretreatment, CI values remained below 1 in the low- and medium-effect fractions (Fa < 0.7). In contrast, CI values exceeding 1 were recorded in the high-effect fractions (Fa > 0.8). Based on the CI analysis, the combined administration of L-Dopa and safranal showed a synergistic effect (CI < 1) at low- and medium-effect levels in reducing 6-OHDA-induced cytotoxicity, whereas an additive or mildly antagonistic effect (CI ≥ 1) was observed at high-effect levels (Figure 4).

### 2.4. Effects of L-Dopa and Safranal, Alone and in Combination, on 6-OHDA-Induced Cytotoxicity and Mitochondrial Membrane Potential in SH-SY5Y Cells

**Figure 5:**
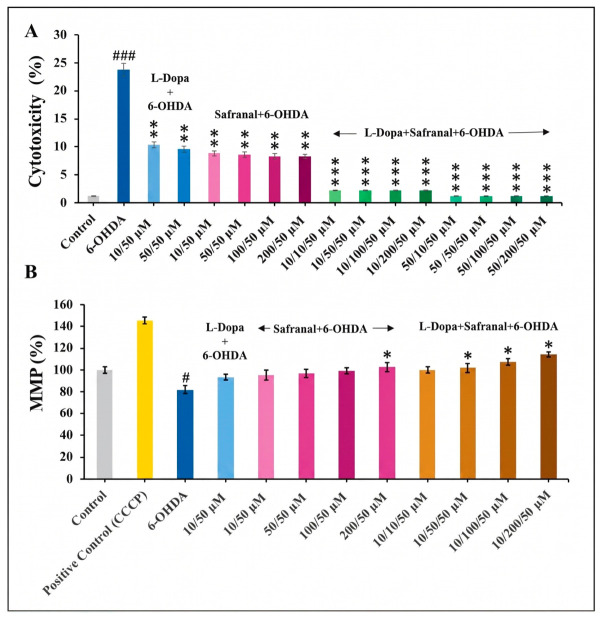
Effects of 4 h pre-treatment with L-Dopa and safranal on (**A**) cytotoxicity, assessed by LDH release, and (**B**) MMP in SH-SY5Y cells exposed to 6-OHDA (50 µM) for 24 h. The data is presented as mean ± SD (*n* = 6). Statistical analysis was performed using one-way ANOVA followed by Tukey’s post hoc test for multiple comparisons. Differences compared with the control group are indicated as # *p* < 0.05, and ### *p* < 0.001, whereas differences compared with the 6-OHDA-treated group are indicated as * *p* < 0.05, ** *p* < 0.01, and *** *p* < 0.001. MMP, mitochondrial membrane potential CCCP, carbonyl cyanide 3-chlorophenylhydrazone.

The cytotoxic effects of L-Dopa and safranal on SH-SY5Y cells were evaluated using lactate dehydrogenase (LDH) analysis. A single administration of 6-OHDA caused significantly higher LDH release than the control condition (*p* < 0.001). Pretreatment with L-Dopa or safranal alone led to significantly lower LDH release compared with 6-OHDA administration (*p* < 0.01). The combined application of L-Dopa and safranal was more effective than either individual treatment. The cytotoxicity levels observed in the combination group were comparable to those in the control group (Figure 5A).

6-OHDA treatment significantly reduced mitochondrial membrane potential (MMP) levels compared with the control group (*p* < 0.05). While pretreatment with either L-Dopa or safranal alone did not significantly increase MMP levels compared with 6-OHDA (*p* > 0.05), pretreatment with 200 µM safranal significantly increased MMP levels (*p* < 0.05). Furthermore, combined pretreatment showed more pronounced effects; specifically, combinations of 10 µM L-Dopa with 50, 100, and 200 µM safranal resulted in significant increases in MMP levels compared with 6-OHDA alone (*p* < 0.05; Figure 5B).

### 2.5. Effect of L-Dopa andSafranal, Alone and in Combination, on 6-OHDA-Induced Caspase-3/7 Activity and Autophagy-Associated Marker Levels in SH-SY5Y Cells

**Figure 6:**
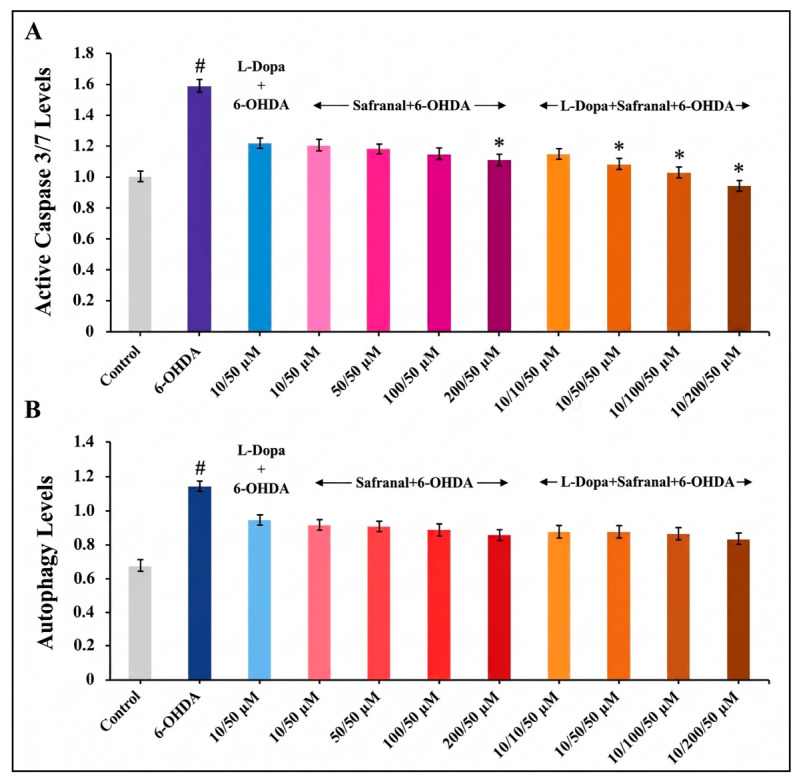
Effects of 4 h pre-treatment with L-Dopa and safranal on (**A**) caspase-3/7 activity and (**B**) autophagy-related marker levels in SH-SY5Y cells exposed to 6-OHDA (50 µM) for 24 h. The data is presented as mean ± SD (*n* = 3). Statistical analysis was performed using one-way analysis of variance (ANOVA) followed by Tukey’s post hoc test for multiple comparisons. Differences compared with the control group are indicated as # *p* < 0.05, while differences compared with the 6-OHDA-treated group are indicated as * *p* < 0.05.

Caspase-3 levels were significantly higher after 6-OHDA treatment than in the control group (*p* < 0.05). However, following pretreatment with L-Dopa and safranal, either individually or in combination, a significant decrease in caspase-3 levels was observed in the 6-OHDA-treated groups compared with the 6-OHDA group (*p* < 0.05). The most pronounced decrease was observed in the group treated with 10 µM L-Dopa and 200 µM safranal compared with the 6-OHDA-only group (*p* < 0.05; Figure 6A).

To investigate the influence of L-Dopa and safranal pretreatments on 6-OHDA-induced neurotoxicity via a cellular response pathway, autophagy-associated marker levels were quantified. Exposure to 6-OHDA led to a significant increase in autophagy-associated marker levels in SH-SY5Y cells compared to the control group (*p* < 0.05). However, both standalone and combined pretreatment with L-Dopa and safranal showed a decrease in these marker levels within the 6-OHDA-treated groups; this reduction was not statistically significant compared to the 6-OHDA-only group (*p* > 0.05; Figure 6B).

### 2.6. Effects of L-Dopa and Safranal on 6-OHDA-Induced mRNA Expression Levles of Circadian Clock Genes in SH-SY5Y Cells

**Figure 7:**
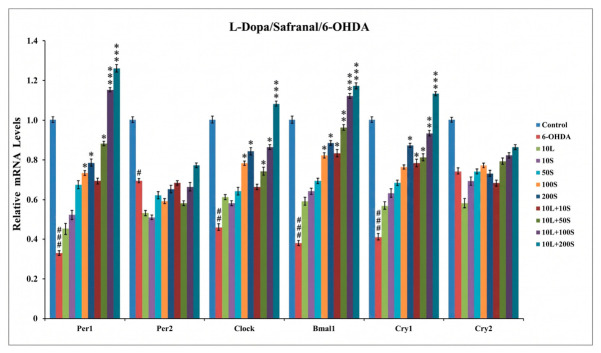
Effects of 4 h pre-treatment with L-Dopa and safranal on the mRNA expression levels of *PER1*, *PER2*, *CLOCK*, *BMAL1*, *CRY1*, and *CRY2* in SH-SY5Y cells exposed to 6-OHDA (50 µM) for 24 h. The data is presented as mean ± SD (*n* = 3). Statistical analysis was performed using one-way ANOVA followed by Tukey’s post hoc test for multiple comparisons. Differences compared with the control group are indicated as # *p* < 0.05, ## *p* < 0.01, and ### *p* < 0.001, whereas differences compared with the 6-OHDA-treated group are indicated as * *p* < 0.05, ** *p* < 0.01, and *** *p* < 0.001. 10L, 10 µM L-Dopa; 10S, 10 µM safranal; 50S, 50 µM safranal; 100S, 100 µM safranal; 200S, 200 µM safranal; 10L + 10S, 10 µM L-Dopa + 10 µM safranal; 10L + 50S, 10 µM L-Dopa + 50 µM safranal; 10L + 100S, 10 µM L-Dopa + 100 µM safranal; 10L + 200S, 10 µM L-Dopa + 200 µM safranal.

The mRNA expression levels of the core circadian genes period circadian regulator 1 (*PER1*), period circadian regulator 2 (*PER2*), circadian locomotor output cycles kaput (*CLOCK*), basic helix-loop-helix ARNT-like 1 (*BMAL1*), cryptochrome circadian regulator 1 (*CRY1*), and cryptochrome circadian regulator 2 (*CRY2*) were quantified using real-time polymerase chain reaction (qPCR). Exposure to 6-OHDA significantly downregulated the expression of *PER1*, *PER2*, *CLOCK*, *BMAL1*, and *CRY1*, whereas there was no significant decrease in *Cry2* expression (*p* > 0.05). While standalone pretreatments with L-Dopa or safranal did not significantly improve the expression of most genes, safranal at concentrations of 100 and 200 µM led to significantly higher expression of *PER1*, *CLOCK*, *BMAL1*, and *CRY1* compared with the 6-OHDA-only group (*p* < 0.05). Notably, combined pretreatments exhibited a concentration-dependent restorative effect. The 10 µM L-Dopa + 10 µM safranal combination significantly increased *BMAL1* and *CRY1* levels; however, higher concentrations (10 µM L-Dopa + 50, 100, or 200 µM safranal) led to more comprehensive improvements, significantly increasing *PER1*, *CLOCK*, *BMAL1*, and *CRY1* mRNA levels. However, even at the highest combination concentration (10 µM L-Dopa + 200 µM safranal), increases in *PER2* and *CRY2* expression remained statistically insignificant (*p* > 0.05; Figure 7).

## 3. Discussion

In the present study, we investigated the potential neuroprotective effects of L-Dopa and safranal, with particular emphasis on cellular responses and circadian-related mechanisms in an *in vitro* PD model. MTT analysis showed that 6-OHDA significantly reduced SH-SY5Y cell viability starting at 10 µM, reaching approximately 70% cytotoxicity at 50 µM. This finding confirms the well-established neurotoxic effects of 6-OHDA in *in vitro* PD models [19,20,21]. Higher concentrations (75–100 µM) resulted in pronounced cytotoxicity, further supporting previous findings in similar *in vitro* PD models [22,23]. Based on these observations, 50 µM 6-OHDA was selected as a concentration that reliably induces neurotoxicity while preserving partial cell viability, consistent with commonly used experimental conditions in *in vitro* PD models [24,25].

Furthermore, 6-OHDA-induced cytotoxicity was quantitatively analyzed using concentration–response modeling and IC_50_ determination. The calculated IC_50_ value (~107 µM) closely aligns with ranges reported in the literature for comparable experimental conditions, supporting the reliability of the model [26,27]. Collectively, these findings confirm that 6-OHDA induces robust, concentration-dependent cytotoxicity in SH-SY5Y cells and support its suitability as a neurotoxic agent for *in vitro* PD modeling.

After establishing the 6-OHDA-induced *in vitro* PD model, we evaluated the effects of L-Dopa and safranal on SH-SY5Y cell viability under both basal and neurotoxic conditions. Neither compound affected cell viability when applied alone, indicating no intrinsic cytotoxicity at the tested concentrations. In contrast, both L-Dopa and safranal modulated cell viability under 6-OHDA-induced stress in a concentration- and time-dependent manner. While L-Dopa showed moderate effects, safranal produced more pronounced improvements, particularly at low to moderate concentrations. Similar concentration-dependent responses have been reported for bioactive compounds in 6-OHDA *in vitro* models, where beneficial effects are typically observed within a defined dose range [14,15,16,28,29,30,31,32]. These findings suggest an optimal concentration window beyond which the efficacy of these compounds may decline.

Short-term pretreatment indicated a synergistic interaction between L-Dopa and safranal (CI < 1), reflected by a greater reduction in 6-OHDA-induced cytotoxicity compared with individual treatments. However, this pattern was not consistent across all effect levels. At higher effect levels (Fa > 0.8), CI values exceeded 1, suggesting a shift toward additive or mildly antagonistic interactions [33]. This effect level-dependent transition suggests that cellular responses vary across different degrees of neurotoxic stress. At lower to moderate effect levels, L-Dopa and safranal may exert complementary protective effects—such as attenuation of oxidative stress, preservation of mitochondrial function, and suppression of apoptotic signaling—resulting in synergistic interactions. In contrast, at higher concentrations, cellular homeostatic systems may approach a threshold beyond which protective mechanisms become saturated or dysregulated, thereby limiting the capacity of the combination to sustain synergistic effects.

This effect level-dependent transition may be associated with several dose-dependent biological processes. Although safranal is widely recognized for its antioxidant properties, it may, under certain conditions, exhibit pro-oxidant-like behavior, potentially disrupting cellular redox balance and increasing intracellular oxidative burden. This observation is consistent with a hormetic dose–response relationship, in which low to moderate doses promote adaptive cellular responses, whereas higher concentrations lead to inhibitory or cytotoxic effects [34,35]. Furthermore, increasing concentrations may reduce target specificity, resulting in non-specific interactions that mask protective signaling pathways. At these higher levels, the compounds may also trigger cellular stress responses, potentially enhancing apoptotic signaling and thereby limiting the overall neuroprotective capacity of the combination. Notably, previous studies have shown that high doses of antioxidant compounds can induce detrimental cellular effects, including increased oxidative burden and premature cellular senescence [36]. A similar dose-dependent pattern may also apply to L-Dopa, as higher concentrations have been associated with increased oxidative burden due to dopamine metabolism [37]. Ultimately, the transition from synergy to additive or mildly antagonistic interactions may reflect the crossing of a critical cellular threshold at which compensatory mechanisms become insufficient. Together, these findings underscore the importance of dose optimization and highlight the existence of a defined therapeutic window for both compounds in combination-based neuroprotective strategies.

The LDH assay findings further confirmed that 6-OHDA induced significant cytotoxicity in SH-SY5Y cells, which is consistent with previous studies demonstrating increased membrane damage and LDH release following oxidative stress-mediated neuronal injury [30,32]. Both L-Dopa and safranal pretreatments significantly reduced LDH release, indicating the attenuation of cell membrane damage, whereas their combined application exerted a more pronounced protective effect. This enhanced protection is consistent with reports showing that antioxidant and neuroactive compounds can synergistically reduce cytotoxicity by limiting oxidative stress and apoptosis in 6-OHDA-based *in vitro* models [38,39].

The 6-OHDA-induced loss of MMP is a well-established early marker of dopaminergic neurotoxicity in SH-SY5Y cells [14,40,41]. While L-Dopa or safranal alone showed limited effects on MMP restoration, direct evidence regarding the mitochondrial actions of safranal remains limited; however, its antioxidant properties suggest a potential role in suppressing oxidative stress and mitigating mitochondrial dysfunction. Higher concentrations of safranal and, more prominently, their combination, significantly improved mitochondrial function, indicating enhanced protection against mitochondrial depolarization. This effect is consistent with previous reports demonstrating that certain bioactive compounds (e.g., luteolin-7-O-glucoside, loliolide, and vitamin K2) preserve MMP and reduce apoptosis in 6-OHDA models, suggesting that the combined action of L-Dopa and safranal may contribute to the maintenance of mitochondrial integrity [1,32,40].

6-OHDA induces dopaminergic neurotoxicity, primarily through reactive oxygen species overproduction, mitochondrial dysfunction, and activation of the intrinsic apoptosis pathway, resulting in increased caspase-3 activity [40,42,43,44]. Consistent with this, the observed increase in caspase-3/7 activity confirmed effective model induction. Notably, pretreatment with L-Dopa or safranal significantly attenuated this increase, indicating a robust anti-apoptotic effect. This finding aligns with those of previous studies demonstrating that bioactive compounds such as luteolin-7-O-glucoside, bioactive peptides, and plant-derived antioxidants mitigate 6-OHDA-induced apoptosis by preserving mitochondrial function and suppressing oxidative stress-driven caspase activation [14,22,32,43,45,46,47,48]. Importantly, the most pronounced reduction observed in the combined treatment group (10 µM L-Dopa + 200 µM safranal) suggests a potential additive or synergistic interaction, likely mediated through complementary modulation of mitochondrial integrity and redox homeostasis.

Autophagy plays a critical role in maintaining cellular homeostasis in PD models by facilitating the clearance of damaged organelles and protein aggregates. In the present study, 6-OHDA exposure led to a significant increase in autophagy-associated marker levels in SH-SY5Y cells, which may represent a cellular response to oxidative stress. This observation is consistent with previous studies reporting that oxidative stress can trigger autophagy as an early protective mechanism; however, prolonged stress conditions may disrupt autophagic progression and contribute to neuronal damage. Pretreatment with L-Dopa and safranal showed a decrease in these marker levels within the 6-OHDA-treated groups; however, these changes did not reach statistical significance, suggesting that the observed effects may be limited, context-dependent, or dose-sensitive. Importantly, these findings should be interpreted with caution, as the current experimental approach does not allow direct assessment of autophagic flux; therefore, the results reflect alterations in autophagy-associated marker levels rather than dynamic autophagic activity.

The observed downregulation of core circadian clock genes (*PER1*, *PER2*, *CLOCK*, *BMAL1*, and *CRY1*) following 6-OHDA exposure suggests that neurotoxic stress disrupts molecular clock regulation in SH-SY5Y cells, consistent with previous reports of circadian dysfunction in neurodegenerative models [2,49]. In line with this, 6-OHDA can alter the expression of key clock components, such as *BMAL1* and *PER1*, and increase oxidative stress and apoptotic signaling. Mechanistically, inflammatory signaling pathways may contribute to this disruption, as nuclear factor kappa-light-chain-enhancer of activated B cells (NF-κB) has been reported to interfere with BMAL1/CLOCK-mediated transcription through E-box regulation, thereby modulating *PER* and *CRY* gene expression [49,50,51,52].

Pretreatment with safranal, particularly in combination with L-Dopa, resulted in significant upregulation of *PER1*, *CLOCK*, *BMAL1*, and *CRY1* expression, suggesting a partial improvement in circadian clock gene expression levels. This pattern is consistent with previous observations that plant-derived bioactive compounds, including polyphenols, can modulate circadian gene networks and improve cellular resilience under metabolic and oxidative stress [51,52,53]. Such effects have been associated with changes in circadian clock gene expression under stress conditions and reduced apoptotic signaling in stressed cellular systems [51,52]. Dihydroisotanshinone I has been reported to partially restore *BMAL1*, *PER1*, and *CLOCK* expression in a 6-OHDA model and reduce reactive oxygen species levels and caspase-3 activation, supporting a link between circadian regulation and neuroprotection [53]. Importantly, these findings reflect transcriptional changes measured at a single time point and should not be interpreted as evidence of functional circadian rhythmicity. Parameters such as oscillation, phase, and amplitude were not assessed in the present study.

The lack of consistent statistical significance in some treatment groups, particularly with L-Dopa or low-dose safranal treatment alone, may indicate that circadian gene expression is sensitive to dose and combinatorial effects. The limited response observed for *PER2* and *CRY2* further suggests gene-specific regulation or temporal differences in circadian dynamics that may not be fully captured by single time-point measurements. Taken together, these findings suggest that safranal, especially in combination with L-Dopa, may contribute to neuroprotection not only through modulation of oxidative stress and apoptosis but also via partial restoration of circadian clock gene expression, although further time-resolved and mechanistic studies are required to confirm this relationship.

These findings may be interpreted within an integrated biological framework linking mitochondrial dysfunction, apoptotic signaling, and circadian disruption under neurotoxic stress conditions. In the 6-OHDA model, impairment of mitochondrial function, as reflected by the loss of MMP, appears to represent an early and central event that may predispose cells to the activation of intrinsic apoptotic pathways [54], as supported by the observed increase in caspase-3/7 activity. Mitochondria act as multifaceted regulators in this process, where their dysfunction may serve as a critical junction for cell death signaling [55]. This cascade not only compromises cellular viability but may also create a stress environment that could interfere with the stability and regulation of circadian clock machinery through redox-sensitive and inflammatory signaling pathways [56].

In this context, the observed restoration of mitochondrial function and attenuation of apoptotic signaling following L-Dopa and safranal treatment may extend beyond cytoprotection and contribute to the partial normalization of circadian gene expression. Thus, mitochondrial integrity, apoptosis, and circadian regulation may function as interconnected components of a coordinated cellular stress response. The modulation of these interdependent processes by the combined treatment may provide a coherent biological explanation for the observed neuroprotective effects and highlight the multi-layered nature of the cellular response to 6-OHDA-induced neurotoxicity.

This study has several limitations. The use of an *in vitro* SH-SY5Y model may not fully reflect in vivo conditions, and additional analyses—including mitochondrial function, autophagic flux, and time-dependent circadian assessment—are required to further validate these findings. In addition, circadian clock gene expression and autophagy-associated marker levels were evaluated at a single time point. While the results suggest that safranal influences the transcriptional expression of core clock genes and affects autophagy-related signaling, they do not provide direct evidence of functional circadian rhythmicity (e.g., phase or amplitude changes) or dynamic autophagic flux. Future studies incorporating time-course analyses, lysosomal inhibition approaches (e.g., Bafilomycin A1), and real-time reporter systems (e.g., BMAL1-luciferase or tandem fluorescent LC3) would help to clarify the temporal and functional aspects of these processes. In addition, SH-SY5Y cells do not exhibit robust circadian oscillations in the absence of synchronization protocols, which limits their suitability for assessing functional circadian rhythmicity.

## 4. Materials and Methods

### 4.1. Reagents and Chemicals

Safranal (≥90% purity, stabilized; W324707), L-Dopa (L-3,4-dihydroxyphenylalanine, PHR1271), 3-(4,5-dimethylthiazol-2-yl)-2,5-diphenyltetrazolium bromide (MTT), 6-hydroxydopamine (6-OHDA, 162957), Dulbecco’s Modified Eagle Medium (DMEM), and dimethyl sulfoxide were purchased from Sigma-Aldrich (St. Louis, Missouri, USA). Fetal bovine serum (FBS), L-glutamine, penicillin, and streptomycin were obtained from HyClone (Logan, UT, USA). The Cytotoxicity Detection Kit Plus was obtained from Roche Diagnostics GmbH (Mannheim, Germany), the SensoLyte^®^ Homogeneous AMC Caspase-3/7 Assay Kit was obtained from AnaSpec Inc. (Fremont, CA, USA), the Mitochondrial Membrane Potential Assay Kit II was obtained from Cell Signaling Technology (Danvers, MA, USA), and the Autophagy Assay Kit (ab139484) was obtained from Abcam (Cambridge, UK).

### 4.2. Cell Culture

All *in vitro* experiments were conducted using the SH-SY5Y human neuroblastoma cell line obtained from the American Type Culture Collection (ATCC, Manassas, VA, USA; CRL-2266). Cells were cultured in DMEM supplemented with high glucose, 2 mM L-glutamine, 10% FBS, 100 U/mL penicillin, and 100 µg/mL streptomycin. Cultures were maintained at 37 °C in a humidified incubator with 5% CO_2_.

### 4.3. In Vitro PD Model

To model PD *in vitro*, 6-OHDA was used as a dopaminergic neurotoxin. A stock solution of 6-OHDA (20 mM) was prepared in distilled water. SH-SY5Y cells were then exposed to various concentrations of 6-OHDA (5–250 µM) for 24 h. The optimal cytotoxic conditions were determined using the MTT assay.

### 4.4. Cell Viability Assay

Cell viability was assessed using the MTT assay, which measures mitochondrial dehydrogenase activity in viable cells [57]. First, SH-SY5Y cells were pretreated with L-Dopa (at concentrations of 5–500 µM) and safranal (at concentrations of 1–5000 µM) for 4 and 24 h. The medium containing L-Dopa and safranal was then removed from the wells, and the cells were incubated with a medium containing 50 µM 6-OHDA for an additional 24 h. At the end of the incubation periods, cell viability was assessed using the MTT assay by adding 10 µL of MTT solution, prepared at a concentration of 5 mg/mL, to each well and incubating for 4 h. Then, 100 µL of sodium dodecyl sulfate–HCl solution was added to the wells. After an overnight incubation period, the formazan crystals were dissolved, and absorbance values were measured at a wavelength of 570 nm using a SpectraMax M3 device (Molecular Devices, San Jose, CA, USA). Each experimental condition was tested in six technical replicates (six wells per condition in a 96-well plate), and experiments were independently repeated on different days to ensure reproducibility.

### 4.5. Half-Maximal Inhibitory Concentration Analysis

To evaluate the cytotoxic effects of L-Dopa, safranal and 6-OHDA in the PD cell model, cell viability was measured at various concentrations. Dose–response relationships were analyzed using non-linear regression, and IC_50_ values were determined according to the Chou–Talalay method using CompuSyn software (version 1.0 ComboSyn Inc., Paramus, NJ, USA).

### 4.6. Cytotoxicity Assay

Cytotoxic effects were determined by quantifying the release of LDH using the Cytotoxicity Detection Kit Plus (Roche Diagnostics, Mannheim, Germany). SH-SY5Y cells (2 × 10^4^ cells per well) were seeded in 96-well plates and incubated overnight. Following treatment with L-Dopa, safranal, and/or 6-OHDA, 100 μL of the culture medium was transferred to a new 96-well plate, and 100 μL of the LDH reaction mixture was added. After incubation in the dark at room temperature for 30 min, the absorbance was recorded at 490–492 nm.

### 4.7. Caspase-3/7 Activity Assay

SH-SY5Y cells (5 × 10^5^ cells per well) were plated in DMEM containing 10% FBS and antibiotics, after which they were incubated for 24 h. The experimental groups were designated as follows (*n* = 3, technical replicates per condition): Control; 50 µM 6-OHDA; 10 µM L-Dopa; 10, 50, 100, and 200 µM safranal; and 10 µM L-Dopa combined with 10, 50, 100, or 200 µM safranal. The cells were then pretreated with L-Dopa and safranal for 4 h, followed by a 24 h exposure to 6-OHDA. After treatment, cells were washed with cold phosphate-buffered saline (PBS) and lysed using 1× cell lysis buffer containing 1 mM phenylmethylsulfonyl fluoride. The lysates were then centrifuged at 12,000× *g* for 15 min at 4 °C. Protein concentrations were determined using the BCA protein assay kit (Thermo Fisher Scientific, Waltham, MA, USA). Caspase-3/7 activity was measured to assess apoptosis using the SensoLyte^®^ Homogeneous AMC Caspase-3/7 Assay Kit (AnaSpec Inc.) according to the manufacturer’s instructions. From each lysate, 150 µL was combined with 50 µL of substrate solution and incubated in the dark for 1 h. Fluorescence was measured at 380/500 nm (excitation/emission).

### 4.8. Mitochondrial Membrane Potential Assay

MMP, as a proxy for mitochondrial function, was evaluated using the Mitochondrial Membrane Potential Assay Kit II (Cell Signaling Technology). SH-SY5Y cells were seeded at a density of 2 × 10^4^ cells/well and incubated for 24 h. The cells were then pretreated with L-Dopa and safranal for 4 h, followed by a 24 h exposure to 6-OHDA. Then, 10 μL of a 2 μM solution of tetramethylrhodamine ethyl ester was added to each well, after which the cells were incubated for 20 min at 37 °C. After incubation, the cells were washed three times with pre-warmed 1 × PBS to remove any residual fluorescent dye. Then, 100 μL of 1 × PBS was added to each well, and fluorescence was measured at 550/580 nm (excitation/emission) using a SpectraMax M3 plate reader (Molecular Devices, San José, CA, USA).

### 4.9. Autophagy Detection

Autophagic activity was assessed using an Autophagy Detection Kit (ab139484; Abcam). SH-SY5Y cells (2 × 10^4^ cells per well) were seeded in 96-well plates and incubated for 24 h. Treatments included safranal (10, 50, 100, and 200 µM), L-Dopa (10 µM), and combinations of L-Dopa (10 µM) with each concentration of safranal, all applied for 4 h. Once the compounds were added, the positive control group was treated with 6-OHDA (50 µM), rapamycin, and chloroquine. Three separate negative control groups were used. One group was supplemented with dimethyl sulfoxide (DMSO), one with medium containing 10% FBS, and one with serum-free medium. After 24 h, the medium was aspirated, and the cells were washed twice with the assay buffer. Then, 150 µL of Dual Detection Reagent (included in the Autophagy Detection Kit, Abcam, Cambridge, UK) was added, and the cells were incubated for 30 min in the dark. Excess dye was removed with two PBS washes, and 80 µL of fresh buffer was added prior to measuring fluorescence. Autophagic signals were detected at 480/530 nm (excitation/emission).

### 4.10. RNA Isolation and Quantitative Real-Time Polymerase Chain Reaction Analysis

Total RNA was extracted from SH-SY5Y cells following treatment with L-Dopa, safranal, and 6-OHDA using TRIzol reagent (Thermo Fisher Scientific), in accordance with the manufacturer’s protocol. RNA purity and concentration were assessed spectrophotometrically using a NanoDrop 1000 device (Thermo Fisher Scientific), based on absorbance measurements at 260 and 280 nm. Subsequently, one µg of total RNA was reverse-transcribed into complementary DNA (cDNA) using the Transcriptor High Fidelity cDNA Synthesis Kit (Roche Diagnostics), according to the manufacturer’s instructions. The synthesised cDNA samples were stored at −80 °C until further analysis. Quantitative real-time PCR (qPCR) was performed on the LightCycler^®^ 480 Real-Time PCR System (Roche Diagnostics) using the LightCycler^®^ 480 SYBR Green I Master. Target-specific primers were designed using the NCBI Primer-BLAST tool (Primer3 version 2.5.0), and all reactions were conducted according to the manufacturer’s optimised protocols. The primer sequences (5′-3′) used in the qPCR reactions are listed in Table 1. The messenger RNA (mRNA) expression level of beta-actin (β-actin) was used as an internal control to normalise the expression levels of target genes. The thermal cycling conditions were set as follows: an initial denaturation step at 95 °C for 10 min, followed by 45 amplification cycles (95 °C for 10 s, 60 °C for 20 s), and a final cooling step at 40 °C for 30 s. Relative gene expression was quantified using the 2^−ΔΔCT^ method [58,59]. All experiments were performed in triplicate to ensure reproducibility.

### 4.11. Statistical Analyses

All statistical analyses were performed using IBM SPSS Statistics (Version 26.0; IBM Corp., Armonk, NY, USA) [60]. IC_50_ values and CI analyses were calculated using CompuSyn software (version 1.0 ComboSyn Inc., Paramus, NJ, USA), based on the Chou–Talalay method. Relative mRNA expression levels were analyzed using the Relative Expression Software Tool (REST©, 2009 v2.0.13) [59]. For comparisons among multiple experimental groups, one-way analysis of variance (ANOVA) was conducted, followed by Dunnett’s post hoc test for comparisons against a single control group or Tukey’s post hoc test for multiple pairwise comparisons, as appropriate [61,62]. In experiments involving two independent variables (e.g., treatment and time), two-way ANOVA was applied to assess both main effects and their interaction, followed by Tukey’s post hoc test [62]. Student’s *t*-test was used only for direct comparisons between two independent groups, where applicable. Data are presented as mean ± standard deviation (SD). Statistical significance was defined as * *p* < 0.05, ** *p* < 0.01, and *** *p* < 0.001.

## 5. Conclusions

This study evaluated the neuroprotective effects of L-Dopa and safranal, separately and in combination, against 6-OHDA-induced cytotoxicity in SH-SY5Y cells. Both agents showed concentration- and time-dependent effects on key cellular parameters, including cell viability MMP, caspase 3/7 levels, autophagy levels, and circadian rhythm associated genes. Notably, the combined administration of L-Dopa and safranal produced a synergistic protective effect, with co-treatment significantly outperforming monotherapy. In summary, safranal may represent a promising neuroprotective agent capable of influencing key cellular processes in dopaminergic cells. Co-administration with L-Dopa may offer a potential combinational strategy for reducing neuronal damage associated with Parkinson’s disease. Further *in vivo* investigations and mechanistic studies are required to confirm these findings and support the development of combination-based approaches targeting multiple pathological processes in neurodegenerative disorders.

## Figures and Tables

**Table 1:** Gene-Specific Primer Sequences Used in qPCR.

Gene	Forward Primer (5′-3′)	Reverse Primer (5′-3′)
*PER1*	TGGCTATCCACAAGAAGATTC	GGTCAAAGGGCTGGCCCG
*PER2*	GGCCATCCACAAAAAGATCCTGC	GAAACCGAATGGGAGAATAGTCG
*CLOCK*	TGCGAGGAACAATAGACCCAA	ATGGCCTATGTGTGCGTTGTA
*BMAL1*	GGCTCATAGATGCAAAAACTGG	CTCCAGAACATAATCGAGATGG
*CRY1*	CCGTCTGTTTGTGATTCGTG	AAGTTAGAGGCGGTTGTCCA
*CRY2*	GGAGGCTGGTGTGGAAGTAG	CGTAGGTCTCGTCGTGGTTC
*β-actin*	AAGGAGCCCCACGAGAAAAAT	ACCGAACTTGCATTGATTCCAG

## Data Availability

The original contributions presented in this study are included in the article. Further inquiries can be directed to the corresponding author.

## Abbreviations

The following abbreviations are used in this manuscript:

PD	Parkinson’s disease
6-OHDA	6-hydroxydopamine
CI	Combination Index
IC_50_	Half-maximal inhibitory concentration
MTT	3-(4,5-dimethylthiazol-2-yl)-2,5-diphenyltetrazolium bromide
DMEM	Dulbecco’s Modified Eagle Medium
DMSO	Dimethyl sulfoxide
FBS	Fetal bovine serum
ATCC	American Type Culture Collection
PBS	Phosphate-buffered saline
L-Dopa	Levodopa
qPCR	Quantitative real-time polymerase chain reaction
cDNA	Complementary DNA
NF-κB	Nuclear factor kappa-light-chain-enhancer of activated B cells
PER1	Period circadian regulator 1
PER2	Period circadian regulator 2
CLOCK	Circadian locomotor output cycles kaput
BMAL1	Basic helix-loop-helix ARNT-like 1
CRY1	Cryptochrome circadian regulator 1
CRY2	Cryptochrome circadian regulator 2
β-actin	Beta-actin
